# Perceptions of nature, nurture and behaviour

**DOI:** 10.1186/2195-7819-9-13

**Published:** 2013-12-12

**Authors:** Mairi Levitt

**Affiliations:** Department of Politics, Philosophy and Religious Studies, Lancaster University, County South, Lancaster, LA1 4YL UK

**Keywords:** Nature and nurture, Genes and environment, Genes and crime, Behavioural genetics

## Abstract

Trying to separate out nature and nurture as explanations for behaviour, as in classic genetic studies of twins and families, is now said to be both impossible and unproductive. In practice the nature-nurture model persists as a way of framing discussion on the causes of behaviour in genetic research papers, as well as in the media and lay debate. Social and environmental theories of crime have been dominant in criminology and in public policy while biological theories have been seen as outdated and discredited. Recently, research into genetic variations associated with aggressive and antisocial behaviour has received more attention in the media. This paper explores ideas on the role of nature and nurture in violent and antisocial behaviour through interviews and open-ended questionnaires among lay publics. There was general agreement that everybody’s behaviour is influenced to varying degrees by both genetic and environmental factors but deterministic accounts of causation, except in exceptional circumstances, were rejected. Only an emphasis on nature was seen as dangerous in its consequences, for society and for individuals themselves. Whereas academic researchers approach the debate from their disciplinary perspectives which may or may not engage with practical and policy issues, the key issue for the public was what sort of explanations of behaviour will lead to the best outcomes for all concerned.

## Perceptions of nature, nurture and behaviour

Trying to separate out nature and nurture as explanations for behaviour, as in classic genetic studies of twins and families, is now said to be both impossible and unproductive. The nature-nurture debate is declared to be officially redundant by social scientists and scientists, ‘outdated, naive and unhelpful’ (Craddock, 2011, p.637), ‘a false dichotomy’ (Traynor 2010, p.196). Geneticists argue that nature and nurture interact to affect behaviour through complex and not yet fully understood ways, but, in practice, the debate continues^1^. Research papers by psychologists and geneticists still use the terms nature and nurture, or genes and environment, to consider their relative influences on, for example, temperament and personality, childhood obesity and toddler sleep patterns (McCrae et al., 2000; Anderson et al., 2007; Brescianini, 2011). These papers separate out and quantify the relative influences of nature/genes and nurture/environment. These papers might be taken to indicate how individuals acquire their personality traits or toddlers acquire their sleep patterns; part is innate or there at birth and part is acquired after birth due to environmental influences. The findings actually refer to technical heritability which is, ‘the proportion of phenotypic variation attributable to genetic differences between individuals’ (Keller, 2010, p.57). In practice, as Keller illustrates, there is ‘slippage’ between heritability, meaning a trait being biologically transmissible, and technical heritability. This is not simply a mistake made by the media or ‘media hype’ but is, she argues, ‘almost impossible to avoid’ (ibid, p.71).

While researchers are aware of the complexity of gene-environment interaction, the ‘nature and nurture’ model persists as a simple way of framing discussion on the causes of behaviours. It is also a site of struggle between (and within) academic disciplines and, through influence on policy, has consequences for those whose behaviours are investigated. There is general agreement between social scientists and geneticists about the past abuses of genetics but disagreement over whether it will be possible for the new behavioural genetics to avoid discrimination and eugenic practices, and about the likely benefits that society will gain from this research (Parens et al. 2006, xxi). In a special issue of the American Journal of Sociology ‘Exploring genetics and social structure’, Bearman considers the reasons why sociologists are concerned about genetic effects on behaviour; first they see it as legitimating existing societal arrangements, which assumes that ‘genetic’ is unchangeable. Second, if sociologists draw on genetic research it contaminates the sociological enterprise and, third, whatever claims are made to the contrary, it is a eugenicist project (Bearman, 2008, vi). As we will see all these concerns were expressed by the publics in this study. Policy makers and publics are interested in explaining problem behaviour in order to change/control it, not in respecting disciplinary boundaries, and will expect the role of genetics to be considered alongside social factors.^2^

Social and environmental theories of criminal behaviour have been dominant in criminology, and in public policy (Walsh, 2009, p.7). Genetic disorders and mental illness have provided explanations for a small minority of offenders with specific conditions. A 2007 survey of American criminologists found that ‘criminologists of all ideological persuasions view alleged biosocial causes of crime (hormonal, genetic, and evolutionary factors and possibly low intelligence) as relatively unimportant’ compared with environmental causes (Cooper et al., 2010). Sociology textbooks have typically discussed biological theories of criminality only as discredited (Haralambos and Holborn, 2004, Giddens, 2009). Biosocial theories are seen as attractive to ‘agents of social control’ and to be more likely to lead to abusive treatment of offenders. However, with increasing research and public interest in genetics more attention has been paid to biological aspects of crime and to genetic variations within the normal range. Research has focussed on violent and antisocial behaviours which are criminal or may be seen as a precursor to criminal behaviour, for example, antisocial behaviour in young people. Media reports have headlined ‘warrior genes’, ‘the aggressive gene’ and the ‘get out of jail free gene’, all referring to levels of monoamine oxidase A (MAOA) (Lea and Chambers, 2007; Levitt and Pieri, 2009)^3^. Think tanks and ethics groups have considered the ethics and practicalities of genetic testing for behavioural traits (Campbell and Ross, 2004; Dixon, 2005 Nuffield Council on Bioethics, 2002).

An attraction of research into genes and behaviour is the hope that identifying a genetic factor that is correlated with an increased incidence of, say, violent and antisocial behaviour, will point to a way of reducing such behaviour. Fotaki discusses the attraction of biological explanations of inequalities in health based on the assumption that genetic interventions ‘would succeed in addressing the causes of ill health that public health policies cannot.’ (Fotaki, 2011, p.641). The danger is that biological explanations ‘are once more employed for political purposes to explain away the social roots of health inequalities.’ (ibid). Social scientists, and criminologists, have presented biological/genetic explanations of behaviour as dangerous in terms of their potential effect on the individuals or groups identified as genetically at risk. There are obvious dangers of discrimination against, and the stigmatisation of, already vulnerable groups who would be the first to be tested i.e. ‘problem’ families or minority ethnic groups. Discrimination could affect education, employment and family life. The effect of an individual being told s/he has a risk based on a genetic test has been much discussed in relation to health risks (Claassen et al., 2010. While such information could be motivating, because it is personalised, it can also induce a fatalistic attitude that discourages the person from taking preventative measures. Claasen et al. conclude that it is important to identify those vulnerable to the fatalistic impact and to tailor health risk information (ibid p.194). Identifying risk for behaviour, rather than for disease, is likely to be more problematic because of the difficulty of finding preventative measures that are within the individuals’ own control.

..using DNA to assess risk, make a diagnosis or tailor treatments, may weaken beliefs in the efficacy of preventive behaviour and reinforce biological ways of reducing risk, resulting in a preference for medication as opposed to behavioural means to control or reduce risk (ibid, xiv).

Claasen et al.’s comment on genetic tests for health conditions could apply equally to parents given a behavioural risk for their young child from a genetic test, perhaps before any problem behaviour was evident. The test result could weaken parents’ belief that they could take action to prevent/reduce the risk of the behaviour developing in their child and pharmaceutical solutions, as posited by Caspi et al. might not be available (Caspi et al., 2002, xvii). However, it is not necessarily the case that evidence of genetic or biological influence on behaviour leads to more punitive treatment. DeLisi et al. give the example of the use of findings from adolescent brain science in the case of Roper v. Simmons in the US which abolished the death penalty for adolescents. On the basis of the research it was stated that young people under the age of 18 ‘are more vulnerable or susceptible to negative influences and outside pressures including peer pressure’ (DeLisa et al., 2010, p.25) When evidence on genetic traits associated with criminal behaviour has been allowed by courts, mainly in the US, it has so far more often been accepted as a mitigating rather than an aggravating factor in the offenders’ behaviour (Denno, 2009, Farahany and Coleman, 2006).

Environmental explanations of behaviour can, of course, also be presented as deterministic, claiming a closed future for those experiencing poverty and disadvantage. However, it is biological explanations that have caused more concern not only because of the history of eugenics but also because they may be seen as more fundamental, being there from birth, and as harder to change. The public in surveys are reported to see the greatest role for genetic factors in physical features, a lesser role in health conditions and a smaller role still in human behaviour (Condit, 2010, p.619).

## Public perceptions

The model of nature/genes and nurture/environment is still used in behavioural genetics, as well as in popular culture, and has implications for public policy, including the treatment of offenders who claim that a genetic trait has influenced their criminal behaviour. The aim of this research was to explore ideas on the causes of behaviour, particularly violent and antisocial behaviour and examine how respondents use the nature/nurture model. This qualitative research looks at the ways in which lay publics in different age groups conceptualise the factors and influences that made them who they are and their explanations for the behaviour of other people; especially violent behaviour. It was hypothesised that the increased research and media emphasis on the role of genetic factors in health and behaviour might result in an increasing interest in ‘nature’, biology and genes as explanations for behaviour particularly among the young, but, when explaining their own behaviour people might prefer to see themselves as agents with control over their lives. By exploring explanations of behaviour with respondents from different generations, age differences should be apparent.

The views of 78 respondents from 3 generations were gathered by individual interview and questionnaires, using the same open ended questions and responses to two real-life criminal court case studies where environmental or genetic factors had been used by the defence team. Respondents were drawn from a group of retired people participating in an informal ‘senior learners’ programme at Lancaster University, a group of their mainly younger relatives and, in order to recruit more third generation respondents, a group of first year students taking a criminology module. The senior learners group had a programme of talks and discussions and could attend undergraduate lectures. They had, by definition, shown an interest in current issues in a range of fields. There were no educational or age requirements for the group but all the volunteers were retired from paid work and were aged from around 65 years to over 80 years.. They had had similar careers to those popular with social science students; social work, probation, teaching and administrative positions. The senior learners were asked to pass on questionnaires to younger relatives to investigate age differences in attitudes. The first 13 senior learners who responded were interviewed but as only 15 questionnaires were received from their relatives ethical approval was obtained to distribute the same questionnaire to Lancaster University students taking the criminology first year module. Most students were enrolled on social science degrees, including psychology and sociology, and age 18 or 19. While the sample of senior learners and relatives had only a few more women than men, 78 per cent of the students were female reflecting the gender balance on the module as a whole. This makes it difficult to comment on any gender differences in responses. No claims to generalisability are made for this exploratory study. Responses were coded and entered on SPSS and also analysed thematically using Atlas-ti.

The introduction to the interviews and questionnaire was ‘I am interested in your views and ideas on what makes us the people we are; what makes people behave the way they do? What is the influence of nature and nurture?’ The terms, nature and nurture were not used again until the final question. Although the terms were not defined all respondents readily used them with consistent meanings. They identified ‘nature’ with biology, ‘what you are born with’ and genes or DNA and nurture with all aspects of the environment including parenting, socio-economic conditions, the food you eat, culture and other people. Their understanding of environment was therefore similar to that used by genetic researchers; environment as everything that is external to the individual, although they tended to refer more to the social than the biological environment.

A general warm-up question asked whether, in their own family, there was anything they thought of as a ‘family trait’. Then respondents were asked; ‘Imagine a baby swapped at birth and brought up in a completely different family– which influences do you think would be most important – the influence of the birth parents or the influences of the new family- and why?’^4^ The rest of the interview schedule, and the subsequent questionnaire, consisted of open-ended questions.

Respondents were asked how they would explain different kinds of behaviour if they came across a child who is kind and considerate; a young person who displays antisocial and aggressive behaviour adult and an adult with criminal convictions for violence. This was to tap into any differences in general explanations of good and bad behaviour in young people and adults. A quotation about the child killers in the Bulger case being ‘unreformable’ was used to ascertain whether they saw some types of violent behavior, and the actors concerned, as immutable. In order to see how respondents conceptualized the influences of nature/biology/genes and environment/people/experiences in their own lives, respondents were asked to write down ‘what or who made you what you are today’ and any explanation of their responses. Comments were gathered on the introduction of an environmental factor (childhood neglect) by the defence in a violent attack by two young boys in England, and on a genetic factor (MAOA levels) introduced by the defence in an criminal court in Italy. Respondents were asked how they thought such evidence should be dealt with; whether it should affect the degree of blame and whether it should affect criminal responsibility. The final question asked if it mattered ‘for individuals or society’ whether nature or nurture was seen as most important in explaining problem behaviour. Those interviewed were asked if they had any further comments and there was a space for any additional comments on the questionnaire.

This paper focuses on the ways in which respondents employed nature/genes and nurture/environment in their responses as a whole and what other concepts they drew on when explaining behaviour.

Respondents’ explanations of what makes people behave the way they do are discussed through three themes.

1. Nurture is more influential than nature
2. Nature and nurture interact
3. Emphasising nature (but never nurture) can be dangerous

## Theme 1: Nurture is more influential than nature

Whether asked about influences on a baby adopted at birth, on their own lives, on an aggressive child or a violent young person, almost all respondents emphasised nurture. Parents and family were seen as the most important influences for babies and young children, moving to peer group and other relationships and experiences for a young person. The explanation for the violent behaviour of an adult had more to do with the individual and the importance of nurture/environment in explaining behaviour weakened. The quotations below explaining behaviour in a child adopted at birth, a young person and an adult illustrate the widening of influences from infancy through childhood and the onus on adults to take responsibility for themselves.

[a child] The environment in which a child grows up in, particularly the influence and role of the parents shapes how a child will grow up and what sort of adult they will be (77 Student).

[a young person] I believe that upbringing shapes a person’s personality. Provisions of education, lifestyle opportunities and friendship groups all determine ….outlook. You can see evidence in young people at the school I teach at (20 Relative).

Once adult they have to take responsibility for themselves and address whatever has been in their background. An adult can’t turn round and say it’s not my fault (5 Senior Learner).

Participants also saw themselves as shaped by the people surrounding them, starting with their parents, or those who brought them up. Several mentioned the illness and/or death of a parent during their childhood and older respondents talked about separation due to the second world war. Students were especially likely to mention the influence of morals instilled in them by their parents, the core values and discipline that they were taught at home. Educational experiences were important to all. For the senior learners the school leaving age had been age 15, so whether or not they stayed on at school and took public examinations was crucial for their future, and, this decision depended largely on their parents and environment. For the student respondents who had come to university from school, life so far has been ‘kind of set-out’ (41 Student), in the sense that they had progressed through the education system to gain qualifications for university. For their peer group it was normal still to be in education or training at the age of 18.

The lasting effects of early influences were particularly striking among the senior learners, because they were much further removed in years from their childhood. Many related stories about parental influence and also about teachers who taught them at least 50 years ago and had affected them for better or worse. For example a senior learner recalled one of her teachers;

I hated primary school – the teacher in 3rd or 4th year juniors [for ages 9–11] I hated her she was not a nice woman….. I passed to go to the grammar school and it shocked her. She made a derogatory comment – may not have been directed at me but felt it was- about some who should have passed and didn’t and some passing who should not have done…… I always vowed I would never be like that when I was teaching….(11 Senior Learner).

Those who related negative influences presented themselves as active in response, not necessarily at the time but later in their lives. For example a student whose mother had died wrote that ‘it made me more independent’ and another student who was bullied at school wrote that ‘it made me stronger’. The adult had to deal with all the influences (negative or positive) and take control.

## Theme 2: Nature and nurture interact

While respondents’ view of themselves and of a child adopted at birth assigned greater influence to environment this did not mean that they held a simplistic model of, for example 60:40 nurture to nature. In this one question when they were asked to choose one or other as the major influence, almost all chose nurture, as many social scientists might do. However, in open questions and comments more complex interactive models were expressed. Environment/nurture can affect genes/nature and vice versa. No one used the term epigenetics but responses referred to the possibility of environmental influences affecting gene expression, for example;

People with certain predispositions (e.g. to violence) are affected by society, and society affects how their genes are expressed (40 Student).

An older respondent reflects on personal experience of child rearing and asks whether nurture is influenced by nature;

I think the nature nurture debate is very interesting. In my family I can see where my children have their own natures that have developed despite being brought up in the same family with the same boundaries etc. However, as a parent did I alter how I nurture them to take into account their nature? (14 Senior Learner).

This quotation illustrates the inseparability of nature and nurture. The child is developing within the family and the parent is developing parenting strategies informed by previous experiences and by other influences including the reactions of the children.

It was obvious to respondents that both genetic and environmental factors impact on everyone (although the role of genes is not yet understood) and it will be harder for some than for others to behave well because of their genes and environment. These people may need different treatment or extra help if they have committed violent and aggressive crimes but that does not excuse their behaviour. Only in exceptional cases, like insanity, can a young person or adult be said to have no choice but to act in a particular way. It is important that people are seen as responsible while also giving them the help they need. In these two comments the treatment for environmental problems and ‘biology’ are similar; the individual can be helped to modify his/her behaviour.

No, [nature and nurture] both play a part, but they can’t be the explanation for everything. Some people grow up in broken homes and get treated appallingly- yet they seem to understand right + wrong and accept responsibility for their actions. There are too many excuses and we never solve any problems, just make them harder to resolve.......I think if you are sane and you know right from wrong you need to suffer the consequences if you’ve committed a crime, but I do appreciate you may need help psychologically if you have anger issues, for example. If we constantly find reasons to diminish blame from people who have committed heinous acts of crime more people will think they can get away with it and it will cause more harm than good (78 Student).

Some say you can’t fight your biology, but there are social factors that can stop bad behaviour like learned restraint (72 Student).

The desire to leave a space for individual agency may be linked to the finding that emphasising nature, but never nurture, could be dangerous. It is clear that as children grow up they can exercise more control over their environment, although some have more control and choices than others. On the other hand, whatever the individual is born with (genes and nature) is, or seems to be, less malleable which could lead to different criminal justice policies and different social perceptions of the criminal.

## Theme 3: Emphasising nature (but never nurture) can be dangerous for society as a whole as well as for the criminal and victims

The question asked was whether it mattered ‘for individuals or society’ if either nature or nurture was seen as most important in explaining problem behavior. The two most popular answers were that both nature and nurture were needed to explain behaviour, or, that nurture was more important and that there were dangers in emphasising nature. No one in the sample regarded an emphasis on nurture as dangerous or detrimental to the individual or society. On the contrary, emphasising nurture was thought more likely to lead to non-punitive treatment of offenders. There would be attempts to alter future behaviour through improved education and parenting and spreading of knowledge in society about the impact nurture has on young people. Society as a whole would share the blame rather than the individual. As a student put it; ‘society as a whole [would be] open for criticism’ (55 S). An emphasis on nurture was therefore seen as more likely to lead to understanding of problem behaviours and effective treatment, however, the individuals were still to be held responsible for their behaviour.

In contrast there was a mistrust of nature/genetic explanations that again centred on the practical consequences for individuals. It would affect the way criminals were treated by others but could also change their view of themselves. Behaviour would be seen as unchangeable, out of the control of the individual or social action. As a consequence, individual accountability might be removed. The idea that individuals must normally be held responsible for their actions was constantly emphasised (Levitt, 2013).

It does [matter] because [if nurture is emphasised] people will care, parent and look after and raise people with more care. However if it’s proven it is nature, then people may lose the will to live (60 Student).

Several SLs referred to the examination at the end of primary education (the ‘eleven plus’) when explaining why they emphasised environment/nurture rather than nature, or, in this case, innate intelligence. The ‘eleven plus’ examination was used to decide which children would be offered a place at an academically selective grammar school and was based on the idea that intelligence, and future academic achievement, could be accurately measured and predicted at the age of 10 or 11.

‘The 11+ was a nature thing. I did the 11+ − it had an effect. Saying children not going to improve or change. Very embedded in the whole idea of nature – it can’t really be true’ (8 Senior Learner).

An emphasis on nature has practical detrimental consequences for individuals. Their status is fixed, for example as ‘not academic’ or ‘born evil’ and suggests, to them and to others, that their ‘nature’ is unchangeable or very difficult to change by individual or social action.

Yes, [it matters] hugely as position of blame is dependent on whether a person chose to do what they did .....nature suggests no control (35 Student).

Those who thought an emphasis on nature meant people were irredeemable either gave that as a reason not to emphasise nature or to suggest that in fact ‘defects’ of nature could be overcome, as in this comment by a student emphasising the power of education;

Yes it is very important because it helps to understand if people are reformable (nurture) or irredeemable (nature). I believe we are determined by our education and thus with the proper help we can change. In the case of people with major biological defects, education is still a way to get over these obstacles and society should be ready to help these people (38 Student).

It might be thought that offenders themselves would embrace a genetic explanation of their behaviour if this was interpreted, as the respondents feared, as meaning they were not responsible for their crimes. However, a small study of juvenile offenders in the Netherlands found that they gave social explanations of their crimes and most rejected the idea that biology might be a factor. They committed a crime for a specific purpose like to get money or to impress others or they gave environmental reasons such as a deprived background or peer pressure or explained their offences were due to psychological conditions brought on by the use of alcohol and soft drugs (Horstkötter et al., 2012, p.291). Whether they gave goal directed or environmental reasons ‘most of them also state that they had a choice and that it was their choice to commit the crime’ (ibid p.292). As one young offender said in interview;

In the end the person makes the choice himself… The choices I have made also had a share in my past. But in the end I am the one who has made these choices (ibid).

## Genes and environment

Respondents were at ease with the language of nature and nurture which was only used in the introduction to the questionnaire or interview. They readily equated genes with nature and nurture with all sorts of environmental influences. There was an acknowledgement that our understanding of environmental factors is greater than our understanding of genetics but that that would change. Older respondents were more likely to be concerned about such a change.

They're going to be doing a lot more with genetics. Influences policy profoundly and people have to be very careful. It worries me that seen to be [more determining]. The complexities don’t get looked at. If you emphasise environment it is safer from a policy point of view because given that most people don’t know what they are talking about it is safer to see the person as redeemable than to come down on the side of genetics and write people off (3 Senior Learner).

This quotation is typical in its view that nature/genes are seen as determining even though the influences on behaviour are, in reality, complex. Like the studies quoted at the beginning of the article respondents often acknowledged the complexities as nature and nurture interact but separated them when explaining the causes of specific behaviours. Students were less likely to be fearful of genetic explanations of behaviour despite their academic interest in social science. However, the hypothesis that young people might be more likely to be interested in genetic explanations for behaviour was not shown in this small study. The senior learners were more likely to refer to reading on genes and display knowledge of genetics. Older respondents and their relatives more often echoed the sociologists’ concerns about behavioural genetics discussed by Bearman earlier (Bearman, 2008). For those who feared the practical consequences of genetic explanations, like the respondent quoted above, ‘it is safer’ to keep away from them.

Some respondents in all age groups were prepared for advances in genetics to change their understanding of behaviour and prepared for current views of genes/nature as more basic, fixed and unchanging to change too. One of the youngest relatives, in her 20s, emphasised our incomplete knowledge of genetic influences on behaviour as a reason for focussing on nurture ‘at present’;

It is very tricky as we cannot see genes and I am not sure that I totally trust the idea of blaming genes for violent behaviour- maybe the person has a gene for passive behaviour as well. …….In any case we can change nurture but at present we cannot change nature so let’s do one thing at a time (20 Relative).

As respondents in this small study grappled with explanations for their own and others’ behaviour they focussed on the practical consequences leading to a greater concern over explanations based on nature than the more familiar ones based on a complex web of environmental factors. Whereas academic researchers approach the debate from their disciplinary perspectives which may or may not engage with practical and policy issues, the key issue for the public was what sort of explanations of behaviour will lead to the best outcomes for all concerned.

## Endnotes

^1^Behavioural epigenetic research has indicated that life experiences can affect gene expression. While controversial the research suggests the possibility of further complications for the nature-nurture relationship as nurture may be said to shape nature (Buchen, 2010 Powledge, 2011). ^2^Bearman op cit iv. The ESRC Cambridge Network Social Contexts of Pathways into Crime (SCoPiC) promoted multidisciplinary research into the causes of crime and included the E risk longitudinal twin study led by Terri Moffitt which investigated how genetic and environmental factors shape children's disruptive behaviour http://www.scopic.ac.uk Accessed 3 Sep 2013. ^3^ Violent and antisocial behaviour in this longitudinal study was correlated with a common genetic trait (low expression of MAOA) only where the person was severely maltreated in childhood. Behaviour was measured on 4 outcomes; diagnoses of conduct disorder, psychological tests of aggression and anti-social personality disorder and convictions for violent crime. Caspi et al. 2002 (supplementary material). ^4^This initial warm-up question implied that the influences of nature and nurture could be separated and quantified as in common usage both in academic and popular discourses. As discussed respondents were able to express their views more fully (and with more complexity) in the subsequent open questions.
